# Superconductivity in Weyl semimetal candidate MoTe_2_

**DOI:** 10.1038/ncomms11038

**Published:** 2016-03-14

**Authors:** Yanpeng Qi, Pavel G. Naumov, Mazhar N. Ali, Catherine R. Rajamathi, Walter Schnelle, Oleg Barkalov, Michael Hanfland, Shu-Chun Wu, Chandra Shekhar, Yan Sun, Vicky Süß, Marcus Schmidt, Ulrich Schwarz, Eckhard Pippel, Peter Werner, Reinald Hillebrand, Tobias Förster, Erik Kampert, Stuart Parkin, R. J. Cava, Claudia Felser, Binghai Yan, Sergey A. Medvedev

**Affiliations:** 1 Max Planck Institute for Chemical Physics of Solids, Nöthnitzer Straße 40, 01187 Dresden, Germany; 2 Department of Chemistry, Princeton University, Princeton, New Jersey 08544, USA; 3 European Synchrotron Radiation Facility, BP 220, 38043 Grenoble, France; 4 Max Planck Institute of Microstructure Physics, 06120 Halle, Germany; 5 Dresden High Magnetic Field Laboratory (HLD-EMFL), Helmholtz-Zentrum Dresden-Rossendorf, 01328 Dresden, Germany; 6 Max Planck Institute for the Physics of Complex Systems, 01187 Dresden, Germany

## Abstract

Transition metal dichalcogenides have attracted research interest over the last few decades due to their interesting structural chemistry, unusual electronic properties, rich intercalation chemistry and wide spectrum of potential applications. Despite the fact that the majority of related research focuses on semiconducting transition-metal dichalcogenides (for example, MoS_2_), recently discovered unexpected properties of WTe_2_ are provoking strong interest in semimetallic transition metal dichalcogenides featuring large magnetoresistance, pressure-driven superconductivity and Weyl semimetal states. We investigate the sister compound of WTe_2_, MoTe_2_, predicted to be a Weyl semimetal and a quantum spin Hall insulator in bulk and monolayer form, respectively. We find that bulk MoTe_2_ exhibits superconductivity with a transition temperature of 0.10 K. Application of external pressure dramatically enhances the transition temperature up to maximum value of 8.2 K at 11.7 GPa. The observed dome-shaped superconductivity phase diagram provides insights into the interplay between superconductivity and topological physics.

## Abstract (web summary)

Materials which simultaneously exhibit superconductivity and topologically non-trivial electronic band structure possess potential applications in quantum computing but have yet to be found. Here, the authors find superconductivity in MoTe_2_, a material predicted to be topologically non-trivial.

## Graphical abstract

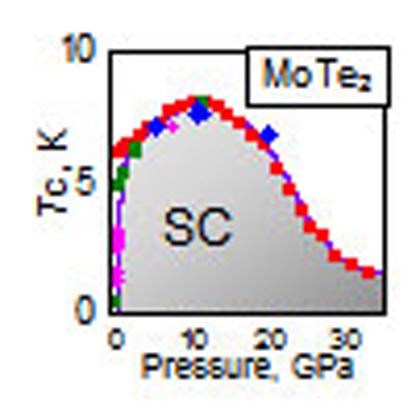

Transition metal dichalcogenides (TMDs) have attracted tremendous attention due to their rich physics and promising potential applications^1^,^2^,^3^,^4^,^5^,^6^,^7^,^8^,^9^,^10^,^11^. TMDs share the same formula, MX_2_, where M is a transition metal (for example, Mo or W) and X is a chalcogenide atom (S, Se and Te). These compounds typically crystallize in many structures, including 2H-, 1T-, 1T′- and T_d_-type lattices. The most common structure is the 2H phase, where M atoms are trigonal-prismatically coordinated by the chalcogenide atoms. These planes then stack on one other with van der Waals gaps inbetween. In contrast, the 1T structure corresponds to octahedral coordination of M. The 1T′ phase is a monoclinic lattice that can be interpreted as a distortion of the 1T phase by the formation of in-plane M–M bonds, resulting in a pseudo-hexagonal layer with zigzag metal chains. Finally, the T_d_ phase is very similar to the 1T′ phase, but the layers stack in a direct fashion, resulting in a higher-symmetry orthorhombic structure. Depending on the synthesis technique, the same composition of MX_2_ can crystallize in a variety of structures with very different electronic properties. For example, MoTe_2_ exists in 2H, 1T′ and T_d_ structures^12^,^13^,^14^, while WTe_2_ has commonly been observed in the T_d_ structure^15^. The 2H and 1T compounds are primarily semiconducting, whereas the 1T′ and T_d_ compounds are typically semimetallic.

Very recently, semimetallic TMDs have attracted considerable attention because of the discovery of salient quantum phenomena. For instance, T_d_−WTe_2_ has been found to exhibit an extremely large magnetoresistance^16^,^17^, pressure (*P*)-driven superconductivity (highest resistive transition temperature *T*_c_≈7 K at 16.8 GPa) (refs 18, 19), and a large and linear Nernst effect^20^. Further, this material has been theorized to constitute the first example of a type-II Weyl semimetal^21^. Moreover, the 1T′-MX_2_ monolayer has been predicted to be a two-dimensional topological insulator^6^.

The discovery of superconductivity in WTe_2_ is apparently contradictory to previous theoretical predictions^22^, which claim that 2H TMDs may become superconducting at high *P*, but the 1T′ phases will not. Thus the investigation of other TMDs for the appearance of superconductivity under pressure is of big interest. Molybdenum ditelluride (MoTe_2_) is unique among the TMDs since it is the only material that can be grown in both 2H and 1T′ forms, allowing for direct examination of this theory. If superconductivity exists in 1T′-MoTe_2_, it may allow the topological edge states to also become superconducting because of the proximity effect in a bulk superconductor. This would open up a new platform for the study of topological superconductivity, which has potential application in quantum computation^23^. Regarding the recently anticipated Weyl semimetal phase in MoTe_2_ (ref. 24), discovery of superconductivity may introduce a new pathway for the exploration of topological superconductivity^25^,^26^,^27^ along with emergent space-time supersymmetry^28^.

Here, we report on the transport properties of the 2H, 1T′ and T_d_ polytypes of MoTe_2_ under various applied *P*. We find that T_d_-MoTe_2_ exhibits superconductivity with *T*_c_=0.10 K, according to electrical resistivity (*ρ*) measurements. Application of relatively low pressures below 1 GPa dramatically enhances the *T*_c_, and a dome-shaped *T*_c_–*P* phase diagram is observed with maximum *T*_c_=8.2 K at 11.7 GPa; this is ∼80 times larger than the ambient pressure value. In contrast, we do not observe any traces of superconductivity in the 2H phase, even when it becomes metallic under *P*. We assume that the extreme sensitivity of the superconductivity to *P* is a consequence of the unique electronic structure. Thus, MoTe_2_ presents the opportunity to study the interaction of topological physics and superconductivity in a bulk material.

## Results

### Structure and transport properties at ambient pressure

**Figure 1:**
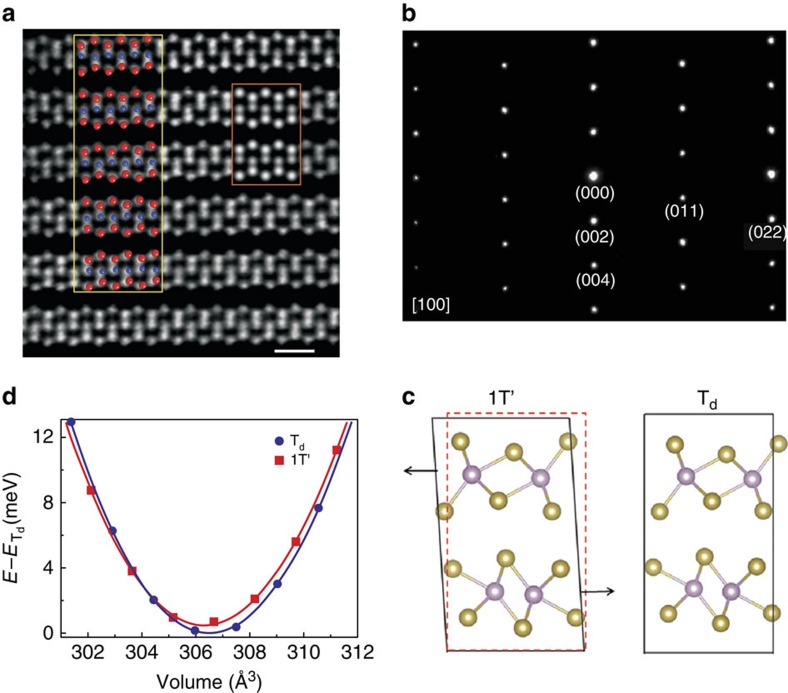
MoTe_2_ crystal structure. (**a**) HAADF-STEM image of 1T′-MoTe_2_ along the [100] zone (scale bar, 0.5 nm). The red rectangle shows HAADF simulated image, and the red and blue spheres in the yellow rectangle represent Te and Mo atoms, respectively. (**b**) Corresponding electron diffraction images. (**c**) 1T′ and T_d_-MoTe_2_ crystal structures. (**d**) Energy-volume dependence for 1T′ and T_d_ phases from DFT calculations.

Prior physical properties measurements, synthesized 1T′-MoTe_2_ samples were structurally characterized (Fig. 1) using single-crystal x-ray diffraction (SXRD) and high-angle annular dark-field scanning transmission electron microscopy (HAADF-STEM). The atomic arrangement of the 1T′ structure was determined using high-resolution HAADF-STEM images and diffraction patterns, as shown in Fig. 1a,b and Supplementary Fig. 1a,b. The crystal structures of 1T′ and T_d_-MoTe_2_ are sketched in Fig. 1c. At room temperature, the crystals exhibit the expected monoclinic 1T′-MoTe_2_ structure, while the SXRD measurements at 120 K indicate a transition into the orthorhombic T_d_ structure. The 1T′-MoTe_2_ structure crystallizes in the *P*2_1_/*m* space group with lattice parameters of *a*=6.320 Å, *b*=3.469 Å, *c*=13.86 Å and *β*=93.917°; these results are consistent with the previously reported structure^12^. The Raman spectra at ambient *P* contain two characteristic peaks (Supplementary Fig. 1c), which are due to the A_g_ and B_g_ vibrational modes of the 1T′-MoTe_2_ structure; this is also in agreement with a previous report^29^. A full structural solution was obtained for the orthorhombic T_d_ phase at 120 K, the refined parameters are given in Supplementary Tables 1 and 2.

**Figure 2:**
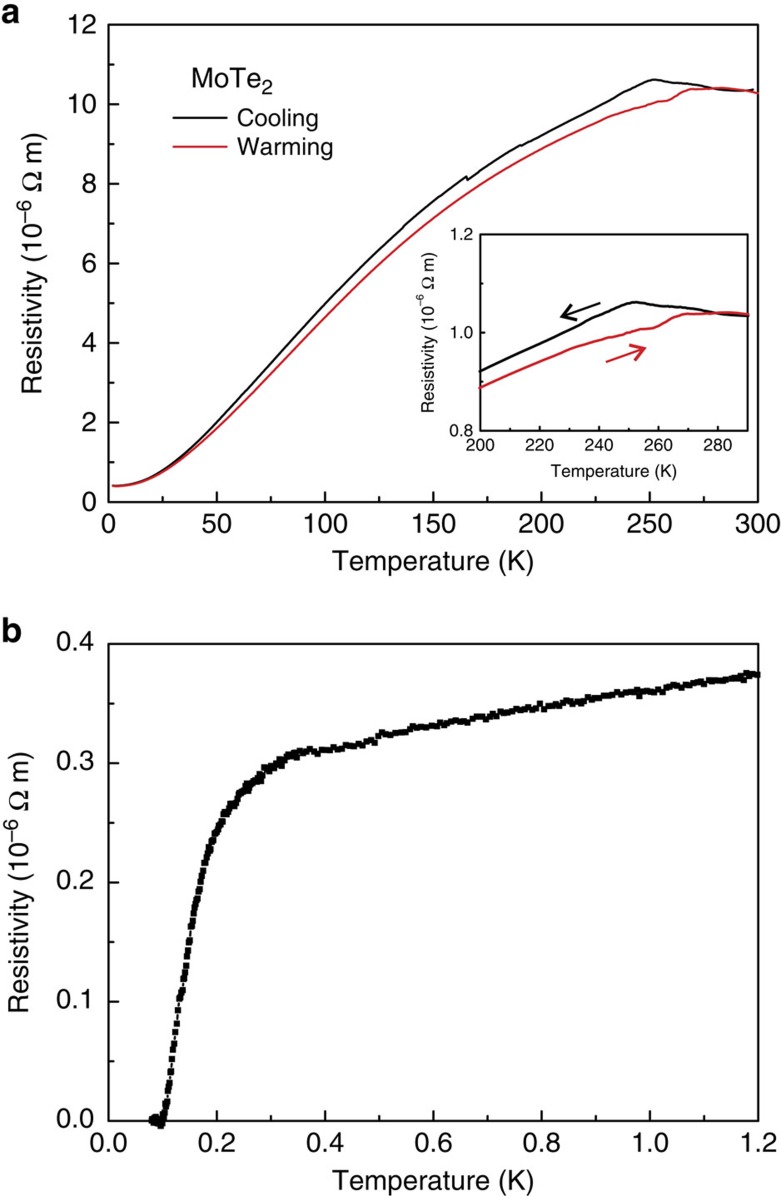
Resistivity of 1T′-MoTe_2_ at ambient pressure. (**a**) Temperature-dependent resistivity at near zero pressure. Inset: anomaly with hysteresis observed at ∼250 K. This hysteresis is associated with the structural phase transition from 1T′-MoTe_2_ to T_d_-MoTe_2_. (**b**) Resistivity detail from 0.08 to 1.2 K. Superconductivity is observed with onset at ≈0.25 K and zero resistance at *T*_c_=0.10 K.

Temperature dependence of electrical resistivity of MoTe_2_ down to a minimum temperature of *T*_min_=0.08 K at ambient pressure is presented in Fig. 2. In contrast to the 2H phase, which displays semiconducting behaviour, 1T′-MoTe_2_ is semimetallic in nature. At zero field, the room-temperature resistivity is *ρ*=1.0 × 10^−5^ Ω m, which decreases to 2.8 × 10^−7^ Ω m at 0.25 K, yielding a residual resistance ratio (RRR) ≈36. At *T*≈250 K an anomaly with thermal hysteresis (Fig. 2a, inset) is observed, which is associated with the first-order structural phase transition from the 1T′ to the T_d_ polytype^14^,^30^. A range of magneto-transport properties has been measured at zero pressure on our MoTe_2_ crystals (Supplementary Figs 2–4 and Supplementary Note 1). From Hall effect measurements, MoTe_2_ shows dominant electron-type transport. Within a single-band model the electron concentration *n*_*e*_ is estimated to 5 × 10^19^ cm^−3^ at 2 K and 8 × 10^20^ cm^−3^ at 300 K (Supplementary Fig. 2), which is close to reported values^29^. In addition, T_d_-MoTe_2_ gradually becomes superconducting below *T*∼0.3 K (the onset of transition), while zero resistance is observed at *T*_c_=0.10 K (Fig. 2b). Note that, although potential superconductivity at∼0.25 K in MoTe_2_ has been briefly mentioned in the literature^31^, no related data have been published.

### 1T′–T_d_ structural transition under pressure

**Figure 3:**
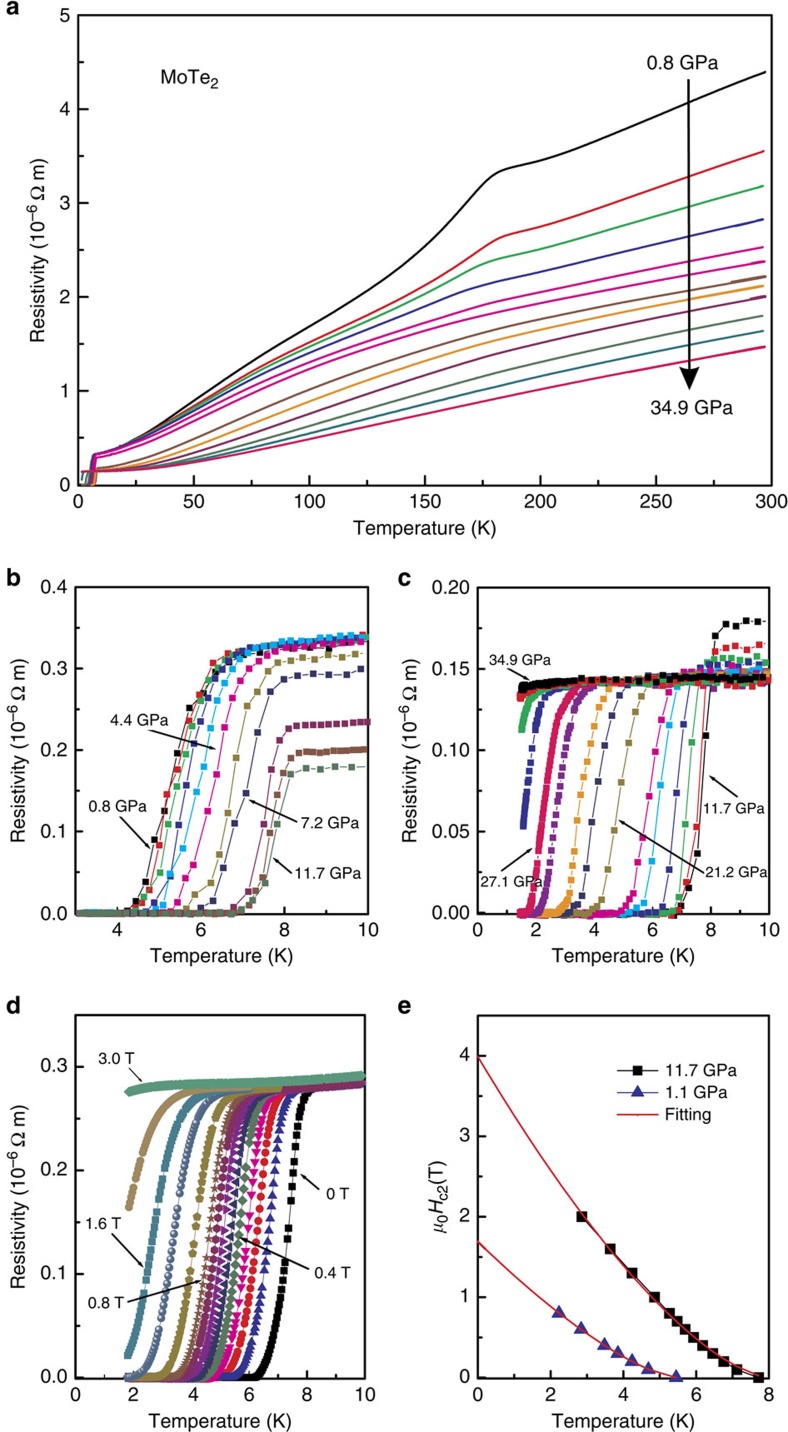
Transport properties of 1T′-MoTe_2_ as a function of pressure. (**a**) Electrical resistivity as a function of temperature for pressures of 0.76−34.9 GPa. The anomaly associated with the structural transition is completely suppressed with increasing pressure. (**b**,**c**) Electrical resistivity as a function of temperature for pressures of 0.7−11.7 and 11.7−34.9 GPa, respectively. Clear electrical resistivity drops and zero-resistance behaviour are apparent. *T*_c_ increases under increasing pressure and a dome-shaped superconducting phase in pressure–temperature space is observed for the maximum superconducting transition temperature corresponding to *T*_c_=8.2 K at 11.7 GPa. (**d**) Temperature dependence of resistivity under different magnetic fields of up to 3 T at 11.2 GPa. (**e**) Temperature dependence of MoTe_2_ upper critical field *H*_c2_. *T*_c_ is defined as temperature at which resistivity drops to 90% of its residual value in normal state. The red curve is the best least squares fit of the equation *H*_c2_(*T*)=*H*_c2_*(1—*T*/*T*_c_)^1+ *α*^ to the experimental data.

**Figure 4:**
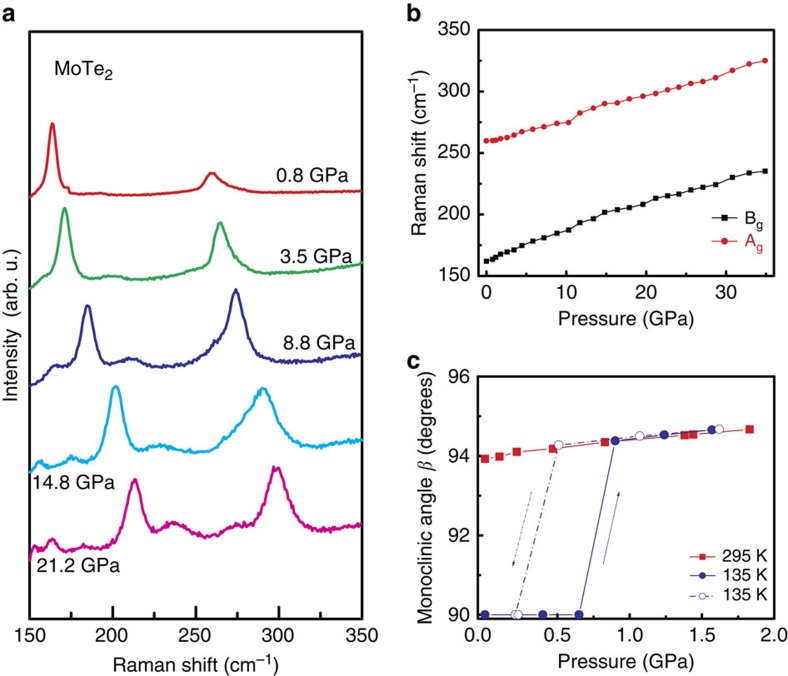
High-pressure Raman spectroscopy and structural studies of 1T′-MoTe_2_. (**a**) Pressure-dependent Raman signals for 1T′-MoTe_2_ at room temperature. The Raman spectra contain two characteristic peaks due to the A_g_ and B_g_ vibrational modes of the 1T′-MoTe_2_ structure. (**b**) Frequencies of A_g_ and B_g_ modes as function of pressure. The frequencies of both vibrational modes increase gradually and continuously as the pressure increases. (**c**) Pressure dependence of the monoclinic angle *β* obtained from SXRD studies. Isothermal compression at room temperature (red filled squares) shows increase of the monoclinic distortion with pressure, whereas reversible orthorhombic T_d_–monoclinic 1T′ transition is observed in isothermal compression (filled blue circles)/decompression (open blue circles) run at 135 K. The values of Raman frequencies in **b** and monoclinic angle in **c** at each pressure are average values obtained from several Raman spectra (XRD patterns) collected from different areas across the sample. The error bars for Raman frequencies in **b** and monoclinic angle in **c** due to s.d. are smaller than the symbols size.

It is well known that high pressure can effectively modify lattice structures and the corresponding electronic states in a systematic fashion. Hence, we measured *ρ*(*T*) for the same 1T′-MoTe_2_ single crystal at various pressure values *P* (Fig. 3). Figure 3a shows the typical *ρ*(*T*) curves for *P* up to 34.9 GPa. For increasing *P*, the metallic characteristic becomes stronger and *ρ* decreases over the entire temperature range. At low pressures, resistance curves exhibit an anomaly at a temperature *T*_s_, associated with the monoclinic 1T′–orthorhombic T_d_ structural phase transition similarly to the ambient pressure data. With pressure increase, the resistivity anomaly becomes less pronounced whereas the temperature of anomaly *T*_s_ is significantly shifted to lower *T* and disappears completely above 4 GPa. Thus, the application of *P* tends to stabilize the monoclinic phase. In addition, the Raman spectra recorded at room temperature under different pressures (Fig. 4a) contain only two characteristic peaks for the 1T′-structure A_g_ and B_g_ modes^29^. The frequencies of both vibrational modes increase gradually with no discontinuities as *P* increases (Fig. 4b) indicating the absence of major structural phase transition in the whole studied pressure range at room temperature. The SXRD data (Fig. 4c and Supplementary Fig. 5) also indicate that application of pressure stabilizes the monoclinic 1T′ structure. Increase of *P* at room temperature results in enhancement of monoclinic distortion (increase of the monoclinic angle *β*). In an isothermal run at 135 K the reversible orthorhombic T_d_ to monoclinic 1T′ transition is observed at ≈0.8 GPa (≈0.4 GPa) at pressure increase (decrease) (Fig. 4c). Thus, application of *P* well below 1 GPa decreases the temperature of structural transition to below 135 K. Furthermore, at *P*≈1.5 GPa, the 1T′ structure remains stable down to at least 80 K. The quantitative discrepancy in the *T*_s_ values derived from structural and resistivity data is most likely due to nonhydrostatic pressure conditions in the resistivity measurements, and the thermal hysteresis since the resistivity curves are recorded with increasing temperatures.

The stability of MoTe_2_ in different phases can be explained using total energy calculations within density-functional theory (DFT). The optimized lattice constants are very close to experimental values for both phases, as shown in Supplementary Fig. 6 and Supplementary Table 3. After evaluating the total energies of the two phases at ambient pressure, we found that the T_d_ phase exhibits slightly lower energy (0.5 meV per formula unit) than the 1T′ phase. This is consistent with the fact that the low- and high-*T* phases are T_d_ and 1T′, respectively, without external pressure. As the 1T′ phase can be obtained by sliding between layers of the T_d_ phase, the former exhibits a slightly smaller equilibrium volume than the latter, as also revealed from the lattice parameters measured via SXRD. As illustrated by the energy-volume profile in Fig. 1d, external pressure will stabilize the 1T′ phase with the smaller volume (and correspondingly higher density) by increasing the shift between neighbouring layers.

### The dome-shaped superconductivity behaviour

**Figure 5:**
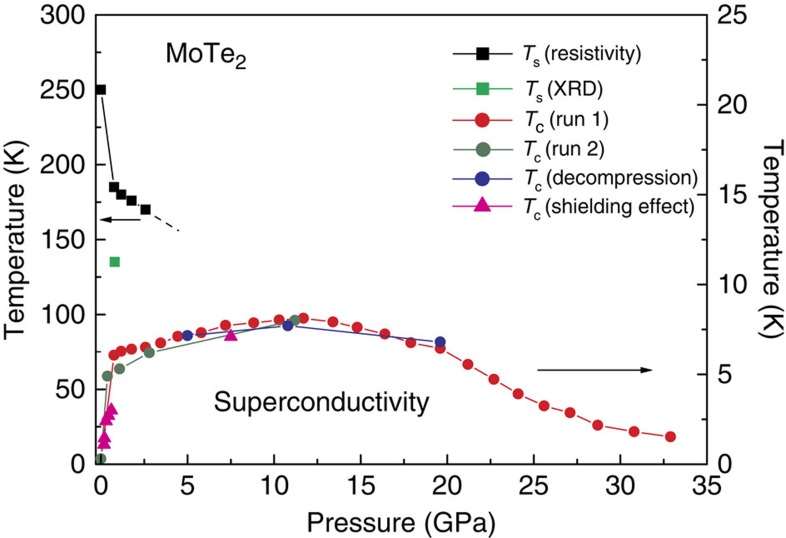
MoTe_2_ electronic phase diagram. The black and green squares represent the structural phase transition temperature *T*_s_ obtained from resistivity and single-crystal synchrotron x-ray diffraction data. The red, blue and olive circles represent the *T*_c_ extracted from various electrical resistance measurements, and the magenta triangles represent the *T*_c_ determined from the magnetization measurements. The error bars deduced from resistivity measurements values of *T*_c_ (red, olive and blue solid circles) due to s.d. of resistivity values (Methods section) are smaller than the symbols size.

Our pressure studies have revealed that the *T*_c_ is very sensitive to pressure. That is, *T*_c_ increases dramatically to 5 K at relatively low pressures below 1 GPa, before beginning a slower increase to a maximum *T*_c_ of 8.2 K at 11.7 GPa (Figs 3b and 5). Beyond this pressure, *T*_c_ decreases and no superconductivity with *T*_c_>1.5 K is found at *P*>34.9 GPa (Fig. 3c). Remarkably, the drastic increase of *T*_c_ at low pressures is associated with a sharp decrease of the 1T′–T_d_ structural phase transition temperature *T*_s_. Subsequently at higher pressures, *T*_c_ still increases to its maximum value with increasing *P* but with significantly lower rate. Our findings demonstrate that the strong enhancement of *T*_c_ at relatively low *P* is associated with suppression of the 1T′–T_d_ structural phase transition. All the characteristic temperatures in the above experimental results are summarized in the *T*–*P* phase diagram in Fig. 5. A dome-shaped superconducting phase boundary is obtained for MoTe_2_, with a sharp slope towards the zero-*P* end of the diagram.

The bulk character of the superconductivity is confirmed by observations of the magnetic shielding effect in the low pressure range and at 7.5 GPa (Supplementary Fig. 7). The onset temperatures of the diamagnetism are consistent with that of the resistivity drop and confirm the drastic increase of *T*_c_ in the low pressure range (Fig. 5). Further, we conducted resistivity measurements in the vicinity of *T*_c_ for various external magnetic fields. As can be seen in Fig. 3d, the zero-resistance-point *T*_c_ under *P*=11.2 GPa is gradually suppressed with increasing field. Deviating from the Werthamer–Helfand–Hohenberg theory based on the single-band model, the upper critical field, *H*_c2_(*T*), of MoTe_2_ has a positive curvature close to *T*_c_ (*H*=0), as shown in Fig. 3e. This is similar to the behaviours of both WTe_2_ (ref. 18) and NbSe_2_ (ref. 32). The experimental *H*_c2_(*T*) data can be described within the entire *T*/*T*_c_ range by the expression *H*_c2_(*T*)=*H*_c2_*(1—*T*/*T*_c_)^1+α^ (refs 18, 33). The fitting parameter *H*_c2_*=4.0 T can be considered as the upper limit for the upper critical field *H*_c2_(*0*), which yields a Ginzburg–Landau coherence length *ξ*_GL_(0) of ∼9 nm. The corresponding data obtained at *P*=1.1 GPa is also shown in Fig. 3e. It is also worth noting that our estimated value of *H*_c2_(0) is well below the Pauli-Clogston limit.

We repeated the high-pressure experiments using different crystal flakes. Similar superconducting behaviour with almost identical *T*_c_ was observed. For comparison with 1T′-MoTe_2_, we also measured *ρ*(*T*) for the 2H-MoTe_2_ single crystal at various pressure values. We found a pressure-induced metallization at 15 GPa (Supplementary Fig. 8), which is consistent with previous theoretical predictions^22^. However, in contrast, we did not detect any signature of superconductivity in the 2H phase for pressures up to 40 GPa.

## Discussion

For MoTe_2_, the superconducting behaviour in the low-*P* region clearly differs from that in the high-*P* region. Under quite low *P*, the sharp increase in *T*_c_ is concomitant with a strong suppression of the structural transition, which is reminiscent of observations for other superconductors with various kinds of competing phase transitions. The drastic increase of the *T*_c_ occurs within the T_d_ phase, which is shown by DFT calculations to be a Weyl semimetal (Supplementary Fig. 9a and Supplementary Note 2) with a band structure around the Fermi level, which is extremely sensitive to changes in the lattice constants^24^,^34^. Thus, one can expect that dramatic structural and electronic instabilities emerge in the low-*P* region, which may account for the strong enhancement of *T*_c_. At higher pressures, the topologically trivial (due to inversion and time reversal symmetry) 1T′ phase (Supplementary Fig. 9b and Supplementary Note 2) remains stable in the whole temperature range. Although within this phase *T*_c_ still continues to increase up to its maximum value, the rate of the increase is significantly lower and this growth is naturally explained by the increase of the electronic density of states at the Fermi level in the 1T′ phase (Supplementary Fig. 9c). Thorough exploration of superconductivity in MoTe_2_ from both experimental and theoretical perspectives is required.

## Methods

### Single-crystal growth

1T′-MoTe_2_ crystals were grown via chemical vapour transport using polycrystalline MoTe_2_ powder and TeCl_4_ as a transport additive^35^. Molar quantities of Mo (Sigma Aldrich 99.99%) were ground in combination with purified Te pieces (Alfa Aesar 99.99%), pressed into pellets and heated in an evacuated quartz tube at 800 °C for 7 days. Crystals were obtained by sealing 1 g of this powder and TeCl_4_ (3 mg ml^−1^) in a quartz ampoule, which was then flushed with Ar, evacuated, sealed and heated in a two-zone furnace. Crystallization was conducted from (*T*_2_) 1,000 to (*T*_1_) 900 °C. The quartz ampoule was then quenched in ice water to yield the high-temperature monoclinic phase. The obtained crystals were silver-gray and rectangular in shape. 2H-MoTe_2_ crystals were grown using a similar method, but without quenching.

### Structural and transport measurements at ambient pressure

The structures of the MoTe_2_ crystals were investigated using SXRD with Mo *K*_*a*_ radiation. To analyse the atomic structure of the material, HAADF-STEM was performed. The dependence of the electrical resistivity *ρ* on temperature *T* was measured using a conventional four-probe method (low-frequency alternating current, Physical Property Measurement System (PPMS), Quantum Design). Temperatures down to 0.08 K were achieved using a home-built adiabatic demagnetization stage. The pulsed magnetic field experiments were conducted at the Dresden High Magnetic Field Laboratory (Helmholtz-Zentrum Dresden-Rossendorf, HLD-HZDR).

### Experimental details of high-pressure measurements

A non-magnetic diamond anvil cell was used for *ρ* measurements under *P* values of up to 40 GPa. A cubic BN/epoxy mixture was used for the insulating gaskets and Pt foil was employed in the electrical leads. The diameters of the flat working surface of the diamond anvil and the sample chamber were 500 and 200 μm, respectively. The initial sample thickness was ≈40 μm. Electrical resistivity at zero magnetic field was measured using the dc current in van der Pauw technique in a customary cryogenic setup (lowest achievable temperature 1.5 K). The resistivity values were defined as an average of five successive measurements at constant temperature. Resistivity measurements in magnetic field were performed on PPMS. Pressure was measured using the ruby scale^36^ by measuring the luminescence from small chips of ruby placed in contact with the sample.

Magnetization was measured on MoTe_2_ (*m*=3.1 mg) in a pressure cell (*m*=170 mg) for *P*≤0.7 GPa and *T*≥0.5 K (Quantum Design Magnetic Property Measurement System (MPMS), iQuantum ^3^He insert). Shielding (after zero-field cooling) and Meißner effect curves (in field-cooling) were recorded.

The high-*P* Raman spectra were recorded using a customary micro-Raman spectrometer with a HeNe laser as the excitation source and a single-grating spectrograph with 1 cm^−1^ resolution. Raman scattering was calibrated using Ne lines with an uncertainty of ±1 cm^−1^.

High-pressure diffraction experiments have been performed at ID09A synchrotron beamline using monochromatic x-ray beam (*E*=30 keV, *λ*=0.413 Å) focused to 15 × 10 μm^2^ on the sample^37^. We used a membrane-driven high-pressure cell equipped with Boehler-Almax seats and diamond anvil design, allowing an opening cone of 64°. The culet size was 600 μm and the sample was loaded together with He as pressure transmission medium into a hole in a stainless steel gasket preindented to ∼80 μm with an initial diameter of 300 μm. Low temperature data were collected in a He-flow cryostat. Single-crystal data have been collected by a vertical-acting *ω*-axis rotation, with an integrated step scan of 0.5° and a counting time of 1 s per frame. Diffraction intensities have been recorded with a Mar555 flat-panel detector. Diffraction data have been processed and analysed with CrysAlisPro-171.37.35 and Jana2006 software. Pressures were measured with the ruby fluorescence method^36^.

### DFT calculations

DFT calculations were performed using the Vienna Ab-initio Simulation Package with projected augmented wave potential^38^,^39^. The exchange and correlation energy was considered at the generalized gradient approximation level for the geometry optimization^40^, and the electronic structure was calculated using the hybrid functional (HSE06)^41^. Spin–orbital coupling was included in all calculations. Van der Waals corrections were included via a pair-wise force field of the Grimme method^42^. In the lattice relaxation, the volumes were fixed while lattice constants and atomic positions were optimized. The pressure was derived by fitting the total energy dependence on the volume with the Murnaghan equation^43^. After checking the *k* convergence, the 24 × 12 × 8 and 7 × 5 × 3 *k*-meshes with Gaussian-type smearing were used for the generalized gradient approximation (Supplementary Fig. 10) and HSE06 calculations, respectively. The band structures, density of states and Fermi surfaces were interpolated in a dense *k*-mesh of 200 × 200 × 200 using the maximally localized Wannier functions^44^ extracted from HSE06 calculations.

## Additional information

**How to cite this article:** Qi, Y. *et al.* Superconductivity in Weyl semimetal candidate MoTe_2_. *Nat. Commun.* 7:10038 doi: 10.1038/ncomms11038 (2016).

## Supplementary Material

Supplementary Information Supplementary Figures 1-10, Supplementary Tables 1-3, Supplementary Notes 1-2 and Supplementary References
