# Supplementary material for: Superconductivity in Weyl semimetal candidate MoTe2
Source: Nat Commun. 2016 Mar 14;7:11038. doi: 10.1038/ncomms11038 (PMC4793082; doi:10.1038/ncomms11038)
Supplement: Supplementary Information — Supplementary Figures 1-10, Supplementary Tables 1-3, Supplementary Notes 1-2 and Supplementary References [file ncomms11038-s1.pdf]

Supplementary Figures

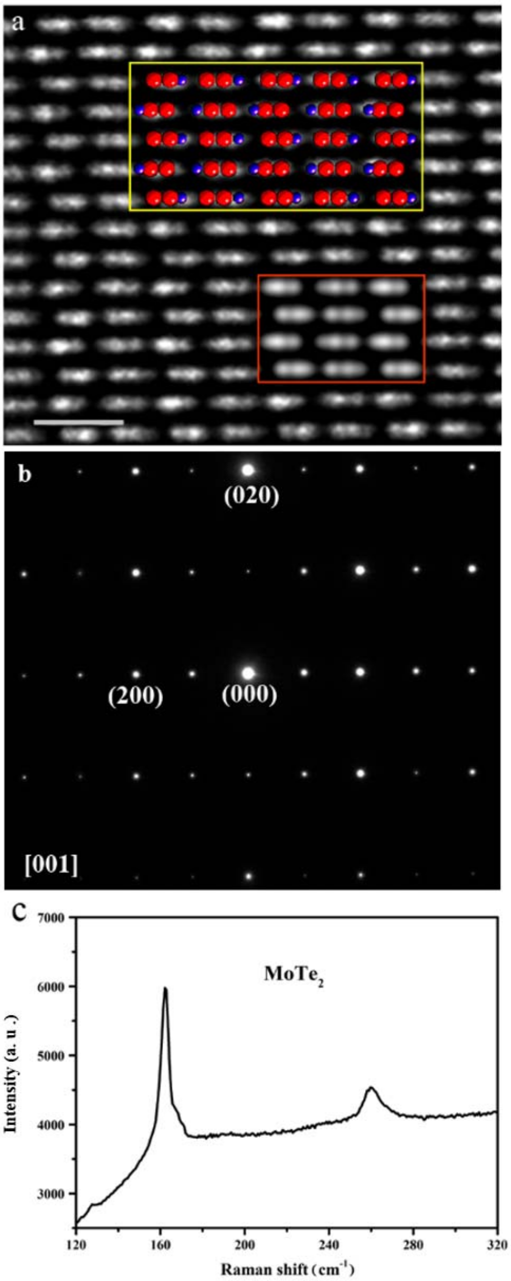

Supplementary Figure 1. Crystal structure of 1T'-MoTe$_2$. (**a**) HAADF-STEM image of 1T'-MoTe$_2$, looking down the [001] zone (scale bar, 0.5 nm). The area indicated by the red rectangle shows HAADF simulated image. The red spheres represent Te atoms and blue spheres represent Mo atoms. (**b**) The corresponding electron diffraction image. **c,** Raman signals for 1T'-MoTe$_2$ at ambient pressure.

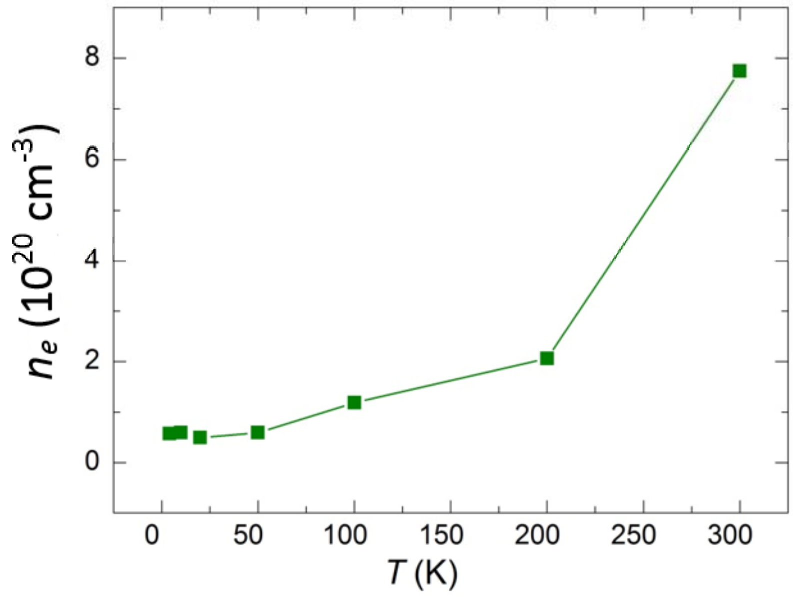

Supplementary Figure 2. Electron charge carrier density of 1T'-$MoTe_2$.

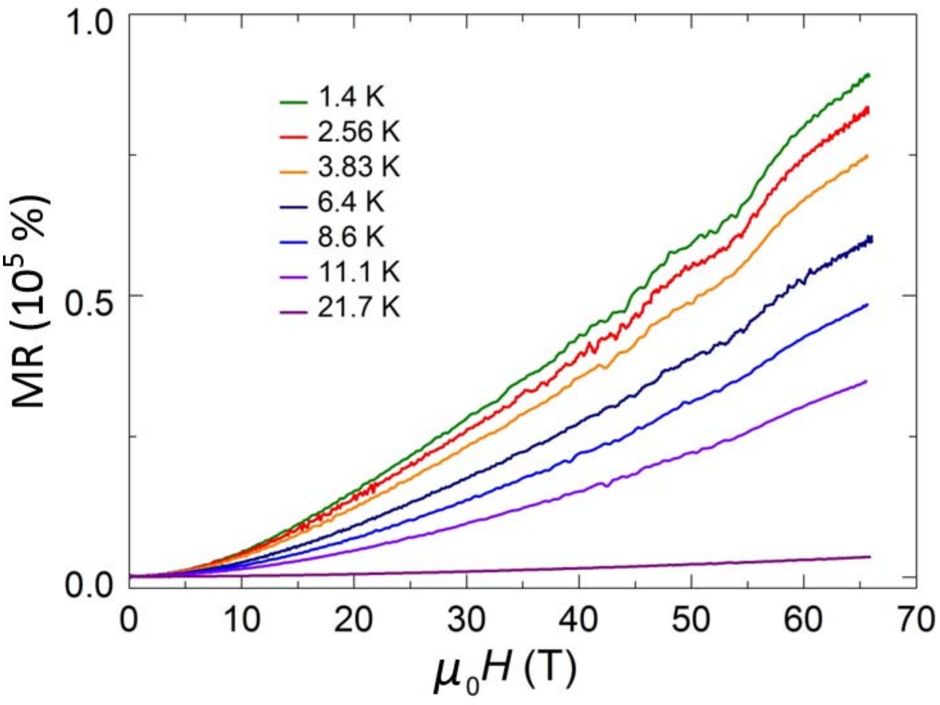

Supplementary Figure 3. Magnetoresistance measured up to a field $\mu_0 H$ = 66 T. Magneto-resistivity superimposed with quantum oscillations known as Shubnikov-de Haas (SdH) oscillations.

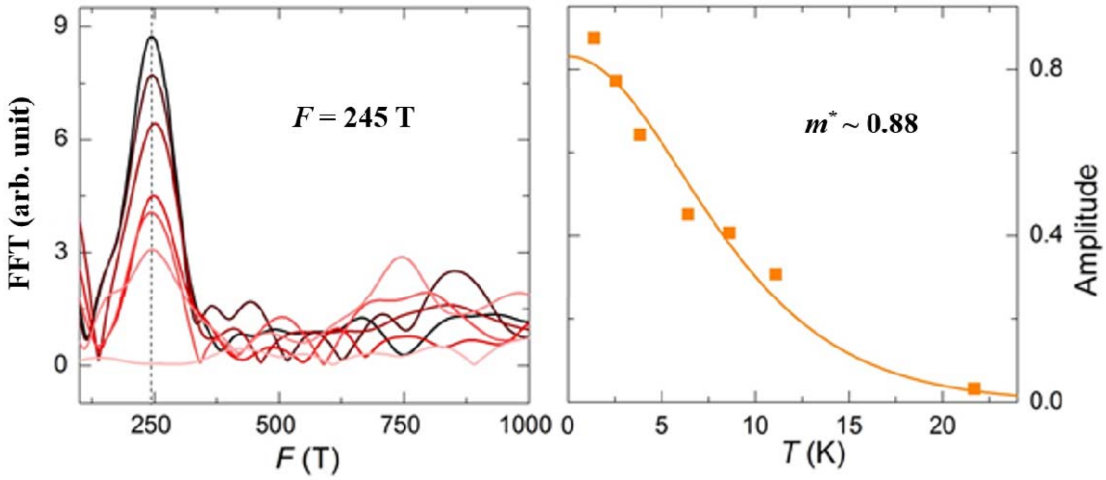

Supplementary Figure 4. Fast Fourier transformation of SdH oscillations. (**a**) The fast Fourier transformation (FFT) of the SdH oscillations. (**b**) Temperature dependent SdH oscillations amplitude.

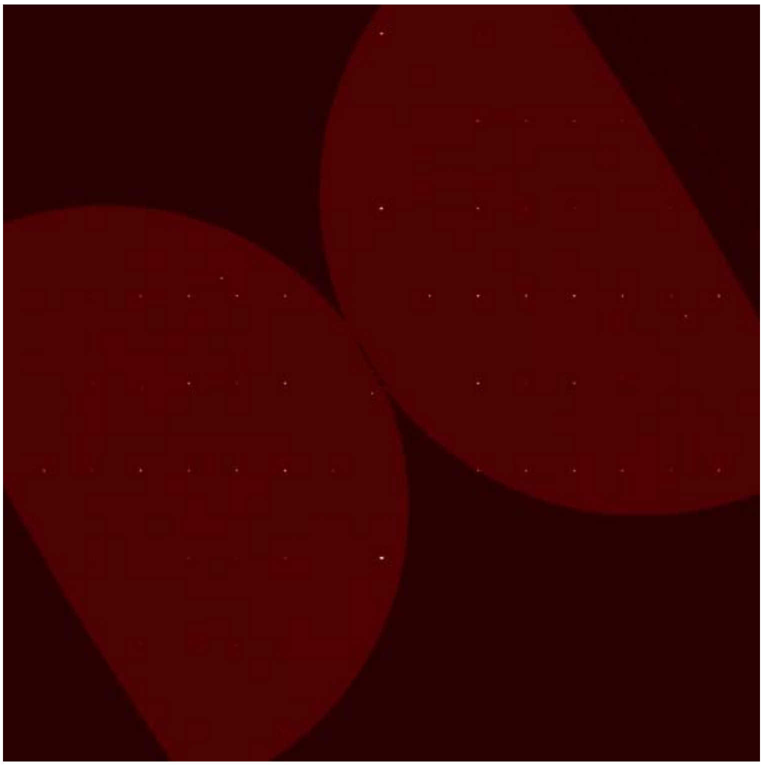

Supplementary Figure 5. Reciprocal space reconstruction of the *hk*0 plane based on single-crystal diffraction data of *β*-$MoTe_2$ collected at pressure 1.56 GPa and temperature 120 K.

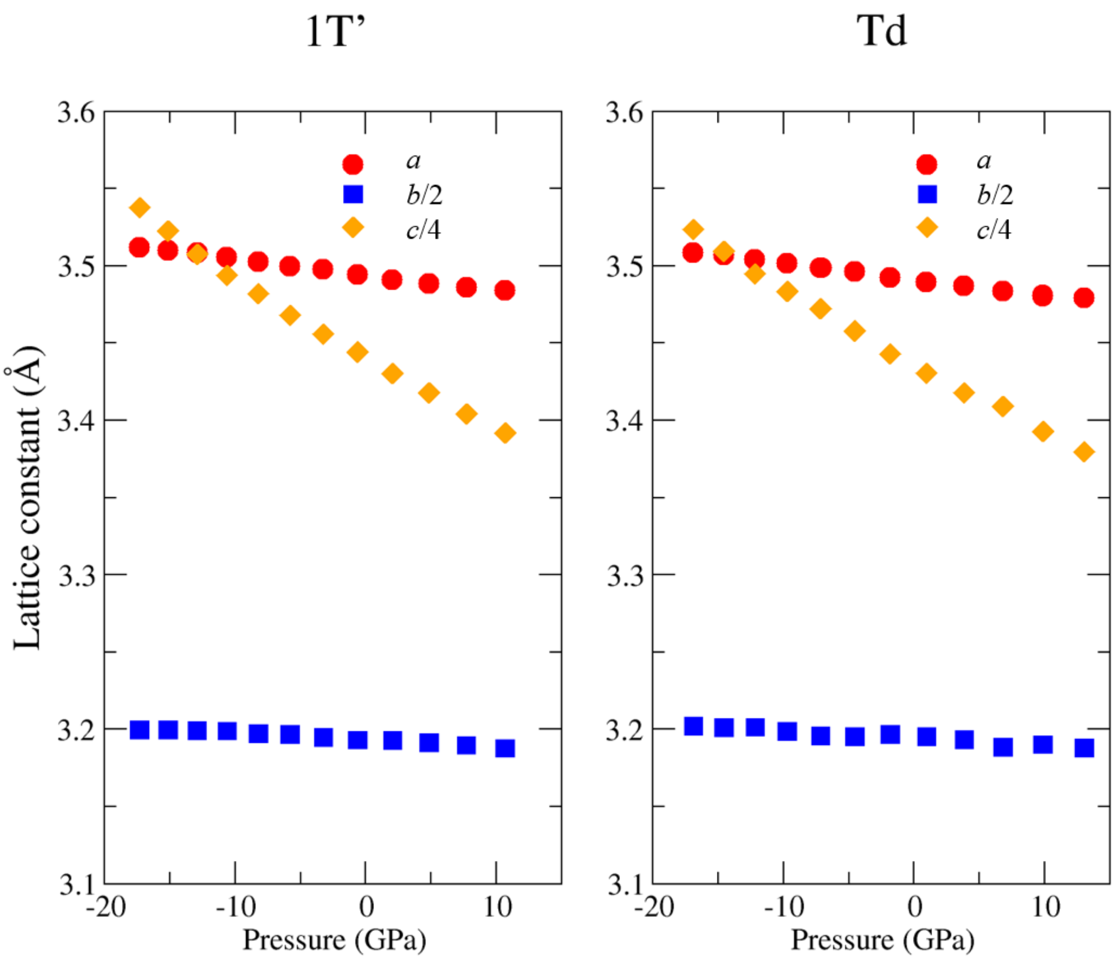

Supplementary Figure 6. Lattice parameters as a function of pressure for T_d and 1T' phases from GGA calculations.

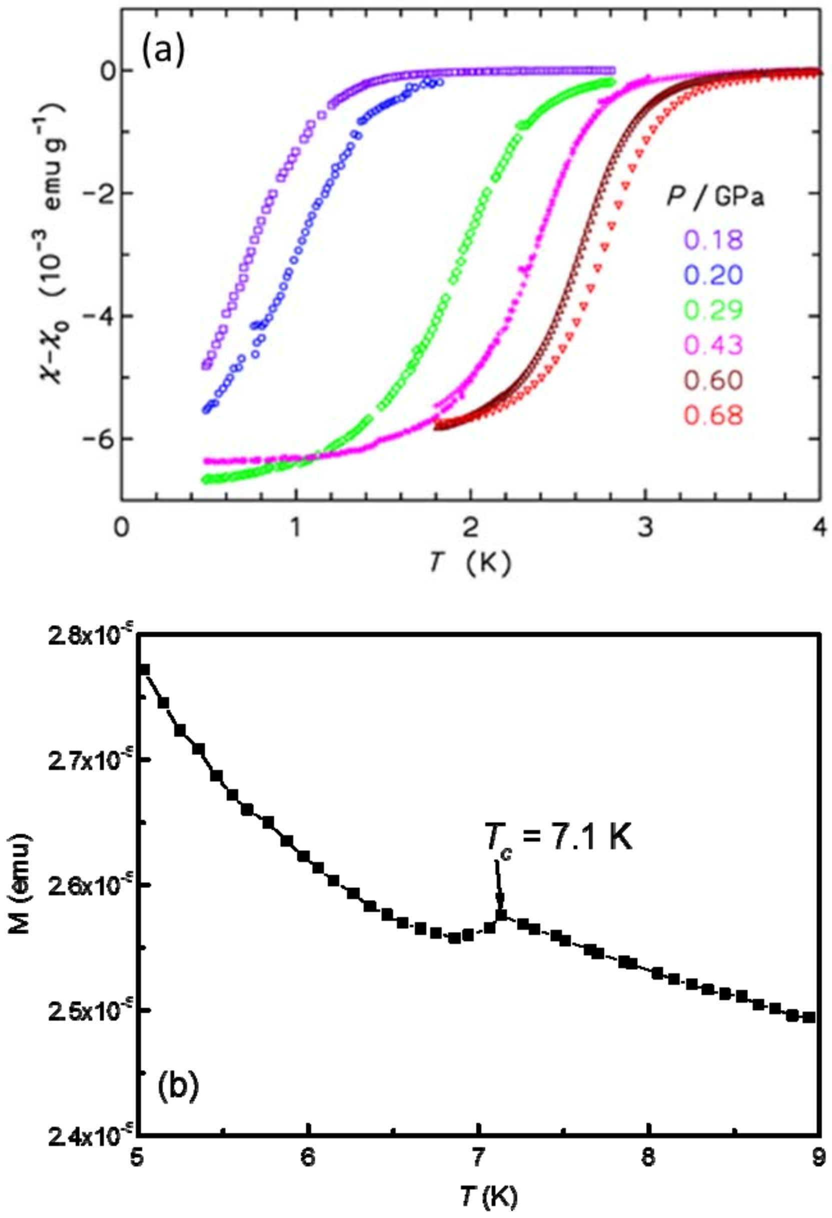

Supplementary Figure 7. Magnetic shielding effect measurements at high pressure. (**a**) The magnetic susceptibility for $P < 0.7$ GPa, (**b**) The raw magnetization signal at $P = 7.5$ GPa for $MoTe_2$, both as a function of temperature in a miniature high-pressure cell[1].

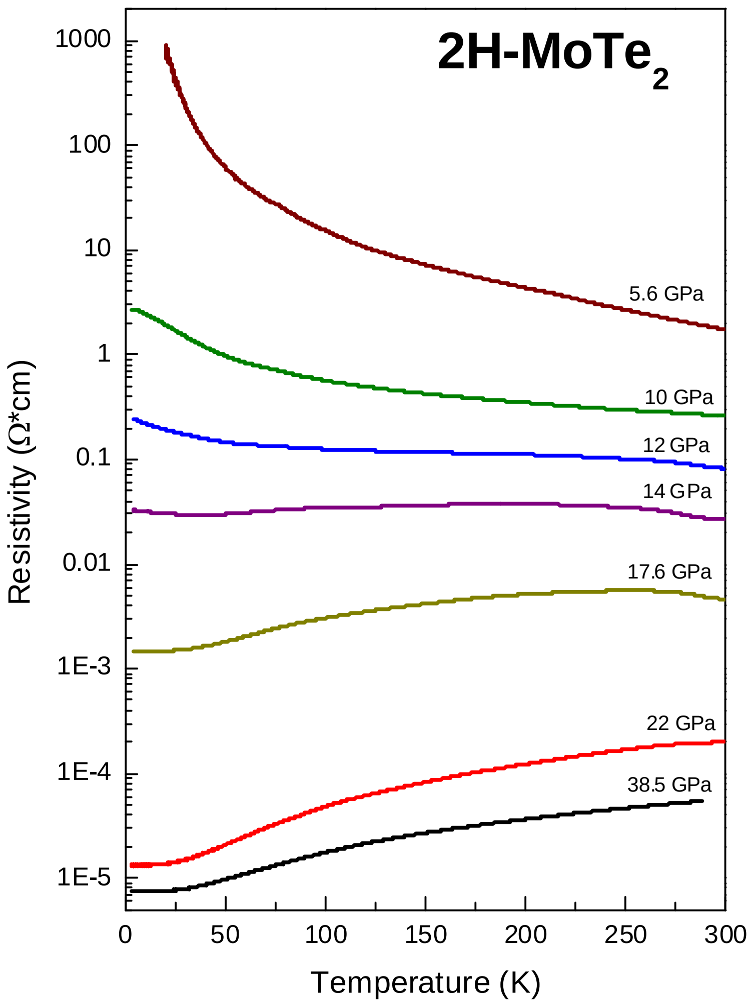

Supplementary Figure 8. Temperature dependence of the resistivity of 2H-MoTe$_2$ under high pressure up to nearly 40 GPa. At pressures below 15 GPa, 2H-MoTe$_2$ remains semiconducting. By 16 GPa, 2H-MoTe$_2$ shows metallic-like behavior the whole temperature range. Thus, 2H-MoTe$_2$ undergoes an insulator-to-metal transition at around 16 GPa, in good agreement with recent theoretical predictions. No signs of superconductivity (T_c > 1.5 K) have been found up to nearly 40 GPa.

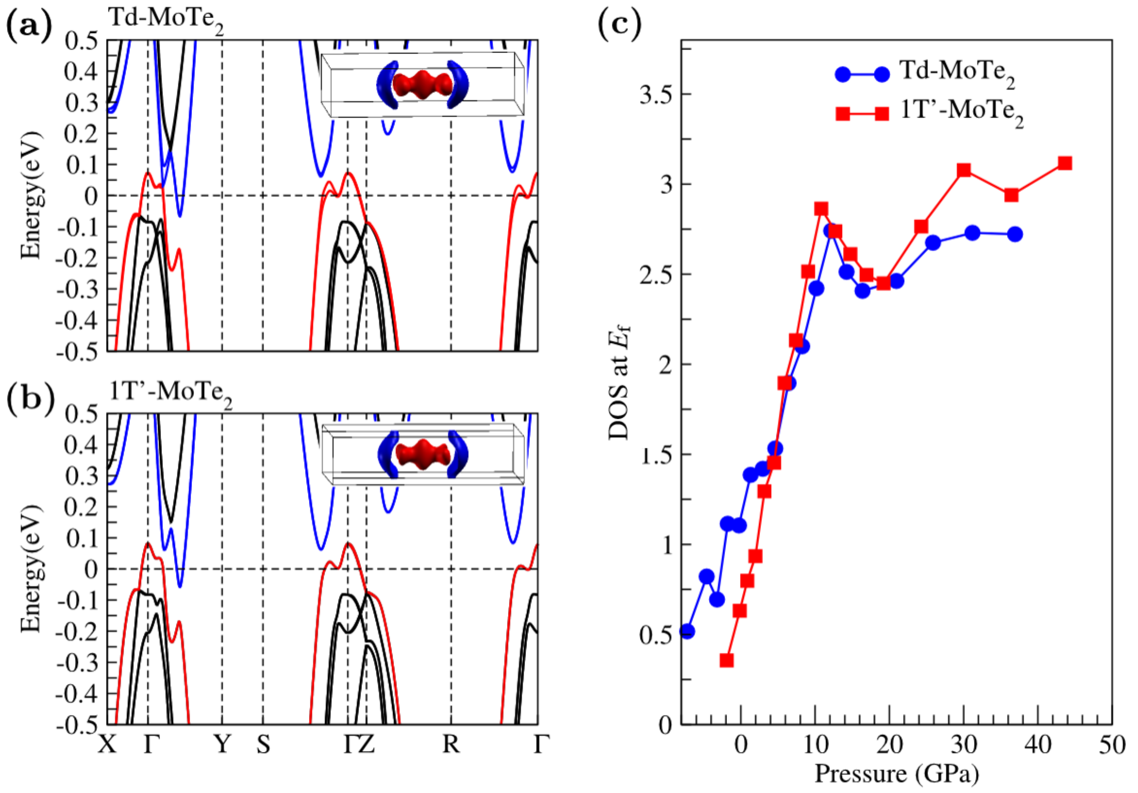

Supplementary Figure 9. Electronic structures obtained from DFT calculations. (**a**) and (**b**) Electronic band structures and Fermi surfaces for $MoTe_2$ in T_d and 1T'-$MoTe_2$ phases respectively with experimental lattice constants. Red and blue Fermi surfaces are hole and electron pockets, respectively. (**c**) The evolution of the density of states (DOS) at the Fermi level as a function of pressure. Please note that a different setting of the elementary cell was used in a previous report[2].

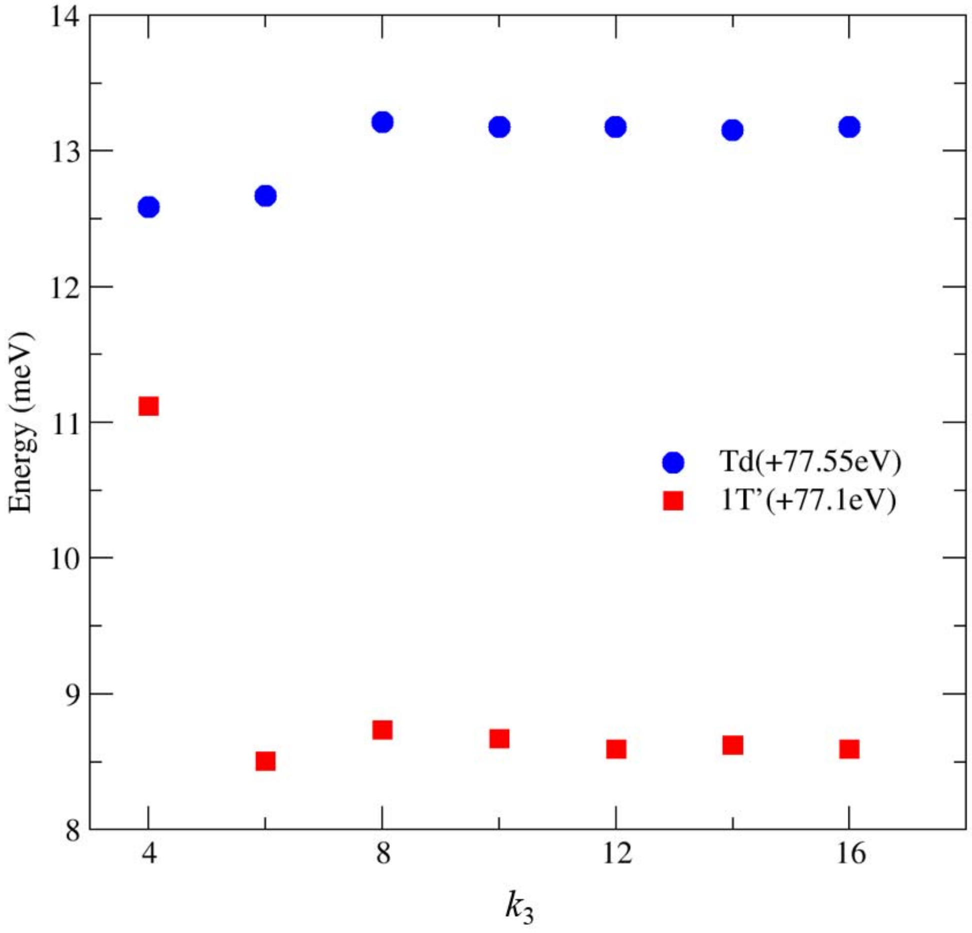

Supplementary Figure 10. *k* grid convergence in DFT (GGA) calculations. We use k_3 to represent the *k*-point density in reciprocal space. The *k* points in three directions have the relation of $3k_1=3k_2/2=k_3$. The convergence arrives at $k_3=8$.

Supplementary Tables

Phase	T_d-$MoTe_2$ (γ-$MoTe_2$)
Symmetry	Orthorhombic, *Pmn2₁* (No. 31)
Cell Parameters (Å)	a = 3.477(2), b = 6.335(3), c = 13.889(6)<br>$\alpha = \beta = \gamma = 90^o$
Wavelength (Å)	Mo $K\alpha$ - 0.71073
V (Å^3)	305.97(20)
Z	4
Calculated Density (g cm^{-3})	7.622(6)
Formula Weight (g mol^{-1})	351.14
Absorption Coefficient (mm^{-1})	22.64
F_{000}	584.0
Reflections	3328
Data/Restraints/Parameters	832 / 38 / 1
Difference e-density (e/ Å^3)	+2.74 to -1.76
R_1 (all reflections)	0.0359
R_1 $Fo > 2\sigma(Fo)$)	0.0312
wR_2(all)	0.076
$R_{int}/R(\sigma)$	0.0812/0.0192
GooF	1.044

Supplementary Table 1. Single crystal structure determination for T_d -$MoTe_2$ (γ-$MoTe_2$), experimental data taken at 120 K.

T_d-$MoTe_2$					
Atom	Wyckoff Site	*x*	*y*	*z*	Occupation
Mo1	2*a*	0	0.6052(2)	0.5003(1)	1
Mo2	2*a*	0	0.0301(2)	0.0147(1)	1
Te1	2*a*	0	0.8626(2)	0.6557(1)	1
Te2	2*a*	0	0.6405(2)	0.1125(1)	1
Te3	2*a*	0	0.2900(2)	0.8593(1)	1
Te4	2*a*	0	0.2160(2)	0.4027(1)	1

Supplementary Table 2. Refined structural parameters for T_d-$MoTe_2$ at 120 K.

	Unit cell Volume (Å^3)		Lattice parameters (Å)							
	Exp.	The.	***a***		***b***		***c***		***β***	
			Exp.	The.	Exp.	The.	Exp.	The.	Exp.	The.
T_d	305.97	306.30	3.477	3.506	6.335	6.360	13.889	13.746	90	90
1T'	303.64	306.48	3.469	3.495	6.330	6.392	13.860	13.741	93.917	93.8576

Supplementary Table 3. Lattice parameters from experimental measurements (Exp.) and DFT relaxation (The.).

Supplementary Note 1

We measured Hall resistivity, $\rho_{xy}(H)$, in a four-probe arrangement at selected temperatures in isothermal field sweeps to derive the Hall coefficient, $R_H(T)$. Importantly, in $MoTe_2$ (1T' at room temperature) $\rho_{xy}(H)$ always exhibits a linear characteristic up to fields of 9 T. The Hall coefficient is negative in the whole temperature range, reflecting electrons as major charge carriers. To calculate the electron charge density, n_e, we used the Drude single-band model[3], $n_e(T) = 1/[e\ R_H(T)]$, where e is the elementary charge. The estimated values of n_e are 5×10^{19} cm^{-3} at 2 K and 8×10^{20} cm^{-3} at 300 K (Supplementary Fig. 2), close to those reported [4].

Magnetoresistance (MR) is defined as the ratio of the change in resistivity due to the applied magnetic field, MR = $\rho_{xy}\ (H)/\ \rho_{xy}(0)$ -1. Here, we measured transverse MR (Supplementary Fig. 3). Resistivity of T_d-$MoTe_2$ is very sensitive to applied field and shows MR of 10^5 % at $T = 1.4$ K without any sign of saturation up to $\mu_0 H = 66$ T. More interestingly, these MR data display Shubnikov-de Haas (SdH) oscillations.

To calculate the amplitude of the SdH oscillations, we fit a 3rd order polynomial to the field dependent resistivity at each temperature. Supplementary Fig. 4a shows the fast Fourier transformation (FFT) of the SdH oscillations that exhibits a cyclotron frequency of the electrons at 245 T. This frequency is equivalent to the periodicity $1/B \approx 0.004$ T^{-1} that corresponds to a cross-sectional area of the Fermi surface $A_F = 0.024$ Å^{-2} from the Onsager relationship $F = \Phi_0/\ (2\pi^2)\ A_F$, where Φ_0 is the magnetic flux quantum. A very small Fermi momentum $k_F = 0.087$ Å^{-1} is obtained supposing a circular cross-section. Further, the cyclotron effective mass of the carriers is determined by fitting the following Lifshitz-Kosevich temperature reduction term[5] to the temperature dependent

SdH oscillations amplitude (Supplementary Fig. 4b).

$$\frac{\Delta\rho_{xx}}{\rho_{xx}}(T) = \frac{14.69m^*T/B}{\sinh(14.69m^*T/B)}$$

The obtained effective mass, m^* is 0.88 m_0, where m_0 is the bare mass of the electron. A lower m^* is observed in few-layer 1T'-$MoTe_2$ specimens[3].

Supplementary Note 2

Consistent with previous reports[6], our calculations show that both T_d-$MoTe_2$ and 1T'-$MoTe_2$ are semimetals at zero pressure, as presented in Supplementary Fig. 9. Since the lattices for two phases are very close, their electronic structures are very similar to each other. As layered compounds, band dispersion is strong in the X-Γ-Y plane and weak along the Γ-Z direction. In the inset of Supplementary Fig. 9a and b, the Fermi surfaces are shown with hole and electron pockets located around the Γ point and the middle of Γ-Y, respectively. One main difference between them is the spin degeneracy. Due to the lack of inversion symmetry, band splitting occurs for spin-up and spin-down states in T_d-$MoTe_2$. In contrast, the 1T' phase has inversion symmetry and all the bands are spin degenerated. At the charge neutral point, we calculated the extreme Fermi surface area of the electron and hole pockets for both two phases. The electron pockets for T_d phase are 0.021 and 0.019 $Å^{-2}$, which correspond to quantum oscillation frequencies of 221 and 200 T, respectively, according to the Onsager relation. Closely, the electron pocket in 1T' phase is 0.0169 $Å^{-2}$, corresponding to a quantum oscillation frequency of 178 T. The extreme Fermi surface area of hole pockets are much larger than 700 T. So we attribute the experimentally observed SdH oscillations frequency (Supplementary Fig. 4) to the electron pockets. The smaller theoretical frequency value, compared with experiment, may indicate that the sample is slightly electron-doped. Since the electron

pocket areas are very close in the two phases, the measured quantum oscillations cannot distinguish them.

In Supplementary Fig. 6 the evolution of the lattice and electronic structures under pressure is shown. Due to van der Waals interactions, the lattice parameter *c* is compressed strongly by the applied pressure, while *a* and *b* only reduce slightly. When the compressive pressure increases from zero to 20 GPa, the density of states (DOS) at the Fermi energy first increases quickly and then decrease slightly after a maximum at around 10 GPa for both T_d and 1T' phases (Supplementary Fig. 9c). This pressure dependence of DOS may be relevant to the dome-shape superconductivity observed in our experiment, since conventional superconductivity is known to be sensitive to the DOS at the Fermi energy.

Supplementary References

1. Alireza, P. L. & Lonzarich, G. G., Miniature anvil cell for high-pressure measurements in a commercial superconducting quantum interference device magnetometer. *Rev. Sci. Instrum.* **80**, 023906 (2009).

2. Sun, Y., Wu, S.-C., Ali, M. N., Felser, C., Yan, B. Prediction of the Weyl semimetal in the orthorhombic $MoTe_2$. *Phys. Rev. B* **92,** 161107 (2015).

3. Ashcroft, N. W. and Mermin, N. D. Solid State Physic (Harcourt, 1976).

4. Keum, D. H. *et al.* Bandgap opening in few-layered monoclinic $MoTe_2$. *Nature Phys.* **11**, 482–486 (2015).

5. Shoenberg, D. Magnetic Oscillations in Metals. Cambridge University Press (2009).

6. Dawson, W. G. & Bullett, D. W. Electronic structure and crystallogrophy of $MoTe_2$ and WTe_2. *J. Phys. C: Solid State Phys.* **20,** 6159-6174 (1987).
